# Supplementary material for: Greater inhibition of female rat binge alcohol intake by adrenergic receptor blockers using a novel Two-Shot rat binge drinking model
Source: Sci Rep. 2024 Jun 18;14:14029. doi: 10.1038/s41598-024-64565-9 (PMC11189554; doi:10.1038/s41598-024-64565-9)
Supplement: Supplementary file 2 — Supplementary Information. [file 41598_2024_64565_MOESM2_ESM.docx]

**Greater inhibition of female rat binge alcohol intake by adrenergic receptor blockers using a novel Two-Shot rat binge drinking model**

Thatiane De Oliveira Sergio^1^, Rebecca Jane Smith^1^, Sarah E. Wean^1^, Eric A. Engleman^1^, Frederic W. Hopf*^1^

Results described in **Fig.3** showed that rats drank approximately the same g/kg alcohol when compared across consumption for 20%, 30%, 40%, or 50% alcohol. To further evaluate if rats were titrating intake to reach a particular level, **Fig.4A-D** showed that consumption level of 20% alcohol correlated, in both sexes, with intake of other alcohol concentrations in most cases, whether examining all Two-Shot intake (**Fig.4A,C**) or Shot-1 intake (**Fig.4B,D**).

However, in strong contrast, Shot-2 20% intake did not correlate with Shot-2 drinking of any other alcohol concentrations in females (**Fig.S1A**; 30%: F_(1,18)_=0.096, *p=*0.7607, R^2^=0.0956; 40%: F_(1,18)_=0.418, *p=*0.5263, R^2^=0.0227; 50%: F_(1,18)_=0.002, *p=*0.9666, R^2^=0.0001) or males (**Fig.S1B**; 30%: F_(1,18)_=0.399, *p=*0.5356, R^2^=0.0217; 40%: F_(1,18)_=0.420, *p=*0.5251, R^2^=0.0228; 50%: F_(1,18)_=1.315, *p=*0.2665, R^2^=0.0681). Thus, these results suggest that, even with briefer intake periods, rats titrated the amount of alcohol consumed for Shot-1 but not Shot-2, regardless of alcohol concentration.

Since rats consumed similar g/kg levels across alcohol concentrations, whether across the 20-minute Two-Shot period or during Shot-1, and females consumed more alcohol for Shot-1 but not Shot-2 (**Fig.3**), titration of intake seemed to primarily occur with Shot-1. In this case, consumption in Shot-2 would not correlate with drinking in Shot-1. Alternately, rats that drank less in Shot-1 might increase intake in Shot-2 to bring intake nearer to an optimal level. However, Shot-2 intake level did not correlate with Shot-1 level for any alcohol concentration in females (**Fig.S1C**; 20%: F_(1,37)_=1.353, *p=*0.2522, R^2^=0.0353; 30%: F_(1,37)_=0.292, *p=*0.5923, R^2^=0.0078; 40%: F_(1,38)_=0.290, *p=*0.5934, R^2^=0.0076; 50%: F_(1,38)_=1.885, *p=*0.1778, R^2^=0.0473). In males (**Fig.S1D**), there was no Shot-1 versus Shot-2 correlation for 20% (F_(1,35)_=0.314, *p=*0.5786, R^2^=0.0089), 30% (F_(1,37)_=1.925, *p=*0.1736, R^2^=0.0495), or 40% (F_(1,35)_=2.524, *p=*0.1211, R^2^=0.0673), although 50% Shot-2 intake was positively correlated with Shot-1 (F_(1,37)_=8.522, *p=*0.0059, R^2^=0.1872). Thus, alcohol titration predominantly occurred within the first 5-minute access period.
